# N-acetylcysteine Provides Cytoprotection in Murine Oligodendrocytes through Heme Oxygenase-1 Activity

**DOI:** 10.3390/biomedicines8080240

**Published:** 2020-07-23

**Authors:** Jie Zhou, Marcia R. Terluk, Lisa Basso, Usha R. Mishra, Paul J. Orchard, James C. Cloyd, Henning Schröder, Reena V. Kartha

**Affiliations:** 1 Center for Orphan Drug Research, Department of Experimental and Clinical Pharmacology, College of Pharmacy, University of Minnesota, 2001 6th Street SE, Minneapolis, MN 55455, USA; zhoux383@umn.edu (J.Z.); mrterluk@umn.edu (M.R.T.); feldh002@umn.edu (L.B.); mishr001@umn.edu (U.R.M.); cloyd001@umn.edu (J.C.C.); 2 Division of Pediatric Blood and Marrow Transplantation, Department of Pediatrics, Medical School, University of Minnesota, 425 East River Parkway, Minneapolis, MN 55455, USA; orcha001@umn.edu; 3 Department of Pharmaceutics, University of Minnesota, 308 Harvard Street SE, Minneapolis, MN 55455, USA; schro601@umn.edu

**Keywords:** N-acetylcysteine, antioxidant, oxidative stress, glutathione (GSH), heme oxygenase-1 (HO-1), oligodendrocytes

## Abstract

Oligodendrocytic injury by oxidative stress can lead to demyelination, contributing to neurodegeneration. We investigated the mechanisms by which an antioxidant, N-acetylcysteine (NAC), reduces oxidative stress in murine oligodendrocytes. We used normal 158N and mutant 158JP cells with endogenously high reactive oxygen species (ROS) levels. Oxidative stress was induced in 158N cells using hydrogen peroxide (H_2_O_2_, 500 μM), and both cells were treated with NAC (50 µM to 500 µM). ROS production, total glutathione (GSH) and cell survival were measured 24 h after treatment. In normal cells, H_2_O_2_ treatment resulted in a ~5.5-fold increase in ROS and ~50% cell death. These deleterious effects of oxidative stress were attenuated by NAC, resulting in improved cell survival. Similarly, NAC treatment resulted in decreased ROS levels in 158JP cells. Characterization of mechanisms underlying cytoprotection in both cell lines revealed an increase in GSH levels by NAC, which was partially blocked by an inhibitor of GSH synthesis. Interestingly, we observed heme oxygenase-1 (HO-1), a cytoprotective enzyme, play a critical role in cytoprotection. Inhibition of HO-1 activity abolished the cytoprotective effect of NAC with a corresponding decrease in total antioxidant capacity. Our results indicate that NAC promotes oligodendrocyte survival in oxidative stress-related conditions through multiple pathways.

## 1. Introduction

Oxidative stress in the central nervous system (CNS) plays a significant role in the pathophysiology of several neurological conditions [1,2]. This leads to the accumulation of reactive oxygen species (ROS), which can damage DNA, proteins, and lipids resulting in cell death [3]. Oligodendrocytes, are a type of glial cells are highly vulnerable to oxidative stress. This can disrupt their maturation and cause cell death, an early event in CNS demyelination and neurodegeneration [4]. Hence, reduction in oxidative stress is being explored as an approach for preventing or slowing down the progression of various neurodegenerative diseases [5,6].

N-acetylcysteine (NAC) is a thiol-containing antioxidant, available both as FDA-approved formulations and dietary supplements. NAC is used as a mucolytic agent in cystic fibrosis and as an antidote for acetaminophen overdose [7,8]. Because of NAC’s apparent successful clinical use in a wide range of diseases, several mechanisms have been proposed for its beneficial antioxidative and anti-inflammatory activity. NAC acts as an oxidative species scavenger and a precursor of L-cysteine for synthesis of the antioxidant glutathione (GSH) [9,10]. Several studies have demonstrated the important roles of GSH in the brain such as cellular redox signaling and intracellular antioxidant defense. On the other hand, the depletion of GSH in brain has been broadly linked to oxidative stress and cellular damage, contributing to neurodegenerative diseases such as Parkinson’s disease (PD) and Alzheimer’s disease (AD) [11]. NAC can increase brain GSH, and thus can be a potential therapeutic strategy for neurodegenerative diseases [12]. In addition, NAC has been involved in the regulation of tissue protective genes that reduce oxidative damage by ROS [13]. Among the cytoprotective proteins, heme oxygenase-1 (HO-1) is an enzyme induced by thiol-containing biomolecules such as lipoic acid and NAC [14,15]. This enzyme catalyzes the oxidative metabolism of heme to form carbon monoxide, biliverdin that gets converted to bilirubin, and free iron [16,17]. Several of these products have antioxidative properties. Studies in AD, PD and multiple sclerosis have shown evidence that in brain oxidative stress leading to neurodegeneration, HO-1 induction exerts neuroprotective effects against oxidative damage [18,19]. Moreover, HO-1-induced glial cell protection was observed in in vivo models of traumatic brain injury and hemorrhage, both of which are associated with oxidative stress [20]. Thus HO-1 mediated signaling may contribute to the cytoprotective mechanism of NAC action, which may be of particular significance during inflammatory processes downstream of oxidative stress. Despite extensive research, the molecular mechanisms underlying NAC benefits remain poorly elucidated. In this study, we have characterized the mechanisms underlying the favorable benefits of NAC in murine oligodendrocytes in conditions of oxidative stress.

## 2. Materials and Methods

### 2.1. Materials

Dulbecco’s Modified Eagle Medium (DMEM) high glucose medium, antibiotic–antimycotic solution (AA), fetal bovine serum (FBS), phosphate buffered saline (PBS), the fluorescent probe CM-H_2_DCFDA and Trypsin-EDTA were purchased from Life Technologies, (Carlsbad, CA, USA); a 7-AAD fluorescent probe from BD Biosciences (San Jose, CA, USA) was used; hydrogen peroxide (H_2_O_2_), sucrose, mannitol ethylene glycol-bis (2-aminoethylether)-*N,N,N′,N′*-tetra acetic acid (EGTA), N-acetylcysteine (NAC) and L-buthionine-(S,R)-sulfoximine (BSO) were purchased from Sigma-Aldrich (St. Louis, MO, USA); HEPES 1M solution from Mediatech (Manassas, VA, USA) was used; Chromium mesoporphyrin IX chloride (CrMP) was from Frontier Scientific (Logan, UT, USA); and, acetonitrile and ammonium formate in the mobile phase was from Thermo Fisher Scientific (Pittsburgh, PA, USA). Stock solutions of compounds were made in PBS buffer (pH 7.4). NAC stock solution (10 mM) in PBS was filtered prior to use.

### 2.2. Cell Culture and Experimental Conditions

The immortalized murine oligodendrocyte cell lines, 158N (normal) and 158JP (Jimpy) were generous gifts from Dr. Ghandour [21]. The 158N and 158JP cell lines were derived from normal and Jimpy mice, respectively, and display features of well-differentiated oligodendrocytes [21]. A mutation in the proteolipid protein PLP/DM20 in Jimpy mice causes premature death of oligodendrocytes and leads to severe CNS demyelination [22]. In comparison to 158N cells, 158JP cells were observed to have significantly higher spontaneous ROS at baseline [21]. Approximately 10^6^ cells were seeded on 75 cm^2^ culture flasks (Corning Inc., Corning, NY, USA) in DMEM high glucose medium supplemented with 5% FBS and 1% AA. Cells were incubated at 37 °C overnight with 5% CO_2_. For all experiments, NAC, at concentrations ranging from 50 µM to 500 µM, was co-incubated with 500 µM H_2_O_2_ for 24 h. For inhibitor studies, 50 µM BSO (a GSH inhibitor) or 30 µM CrMP (a selective inhibitor of HO activity) was added 20 min prior to addition of NAC and H_2_O_2_.

### 2.3. Cell Survival Assays

Cell survival was measured by a colorimetric method using a CellTiter 96 AQueous One Solution Cell Proliferation Assay (Promega, Madison, MI, USA) and a Cell Counting Kit-8 (Dojindo Laboratories, Kamimashiki gun, Kumamoto, Japan) following the manufacturer’s protocols. The absorbance was measured at 490 nm using a microplate reader (Biotek, Synergy 2, Winnoski, VT, USA). The cell survival results were presented as a percentage of control cells.

### 2.4. Evaluation of ROS Production

Evaluation of intracellular ROS was performed by fluorescence-activated cell sorting (FACS) using fluorescent CM-H_2_DCFDA probes. Cells seeded on 24-well plates were harvested and washed twice with PBS, and stained with 1 µM CM-H_2_DCFDA for 5 min. The samples were subsequently washed twice and resuspended in 250 µL of PBS containing 5 µL of 7-AAD fluorescent probes for analysis. The percentage of positive stained CM-H_2_DCFDA of live cells was used as the indicator for ROS levels in different treatment groups. The data were expressed as fold change relative to the control 158N cells.

### 2.5. Determination of Intracellular GSH

Twenty-four hours after treatment, the cells were washed twice with PBS and the harvested cells were lysed using lysis buffer (20 mM HEPES, 1 mM EGTA, 210 mM mannitol and 70 mM sucrose at pH 7.2). The total GSH levels in cell lysates were measured using a validated liquid chromatography- mass spectrometry (LC–MS) method [23]. This was normalized with total cellular protein content quantified using a Quick Start Bradford protein Assay Kit from Bio-Rad (Hercules, CA, USA) according to the manufacturer’s protocol. Further, relative GSH levels expressed as fold change of controls were calculated by dividing the GSH concentration of the treated group by that of untreated control values.

### 2.6. Total Antioxidant Capacity Assay

Total antioxidant capacity (TAC) was evaluated by a colorimetric method using an Antioxidant Assay Kit from Cayman Chemical (Ann Arbor, MI, USA), following the manufacture’s protocol. TAC was expressed as the equivalent Trolox concentration and normalized to the protein concentration, which was determined by Bradford method, as described previously. TAC levels in untreated 158N cells were used as control values and relative TAC levels in treated groups expressed as fold change.

### 2.7. Statistical Data Analysis

The results were presented as mean ± standard error of the mean (SEM) from at least three independent experiments performed in triplicate. Data were analyzed using a two-tailed unpaired Student’s *t*-test and the one-way analysis of variance (ANOVA) with Tukey’s correction for multiple comparisons. A value of *p* < 0.05 was considered statistically significant. All statistical analysis was performed using GraphPad Prism 8 (GraphPad Software Inc., La Jolla, CA, USA).

## 3. Results

### 3.1. NAC Decreases ROS Content in Oligodendrocytes

**Figure 1:**
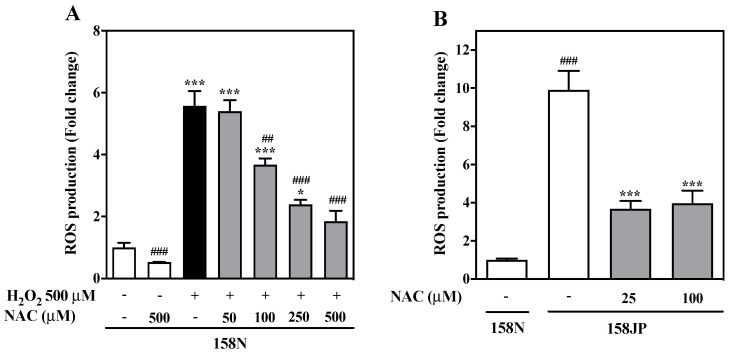
N-acetylcysteine (NAC) decreased reactive oxygen species (ROS) production in oligodendrocytes. (**A**) 158N cells were treated with H_2_O_2_ (500 µM) for 24 h with and without NAC (50 to 500 µM). (**B**) Untreated control 158N cells and 158JP cells treated with NAC (25 µM and 100 µM) for 24 h. The production of ROS was calculated relative to the control 158N cells, and the results were expressed as fold change (**A**,**B**). The ROS levels were measured using a CM-H_2_DCFDA probe by fluorescence-activated cell sorting (FACS). The data were analyzed by one-way analysis of variance (ANOVA) with Tukey’s post-hoc test. *** *p* < 0.001 and * *p* < 0.05 indicate significance between control and treated groups (**A**,**B**). ### *p* < 0.001 and ## *p* < 0.01 show significance comparing (**A**) H_2_O_2_ alone to NAC treatment groups, and (**B**) baseline ROS levels between 158N and 158JP lines.

In order to investigate the effect of NAC on ROS production, 158N cells were incubated either with H_2_O_2_ alone or in combination with increasing concentrations of NAC for 24 h. Untreated cells were used to establish baseline ROS levels in both 158N and 158JP cells, and the values were assumed as controls for each cell line. ROS levels were normalized using the values for 158N cells due to their lower ROS generation rate. As shown in Figure 1A, incubation of 158N cells with the maximum concentration of NAC (500 µM) had no effect on basal ROS production and was similar to the controls (*p* = 0.89). On the contrary, 158N cells exposed to H_2_O_2_ (500 µM) resulted in a 5.6-fold increase in ROS production compared to the control. Co-treatment of 158N cells with both NAC (50 to 500 µM) and H_2_O_2_ significantly decreased ROS production in a concentration-dependent manner (Figure 1A).

Unlike the normal 158N cells, the basal ROS levels of control 158JP cells was approximately 10-fold higher in comparison to control 158N cells (*p* < 0.001, Figure 1B). In 158JP cells, treatment with 25 and 100μM NAC for 24 h significantly attenuated higher constitutive ROS production to 3.6-fold and 4.0-fold, respectively (*p* < 0.001). Thus, there was no concentration-dependent decrease in intracellular ROS with increasing NAC concentration from 25 µM to 100 µM. Our results show that NAC can remarkably decrease H_2_O_2_-induced oxidative stress in 158N cells and the inherent high levels of oxidative stress in 158JP cells.

### 3.2. NAC Improves 158N Cell Survival in Oxidative Stress

**Figure 2:**
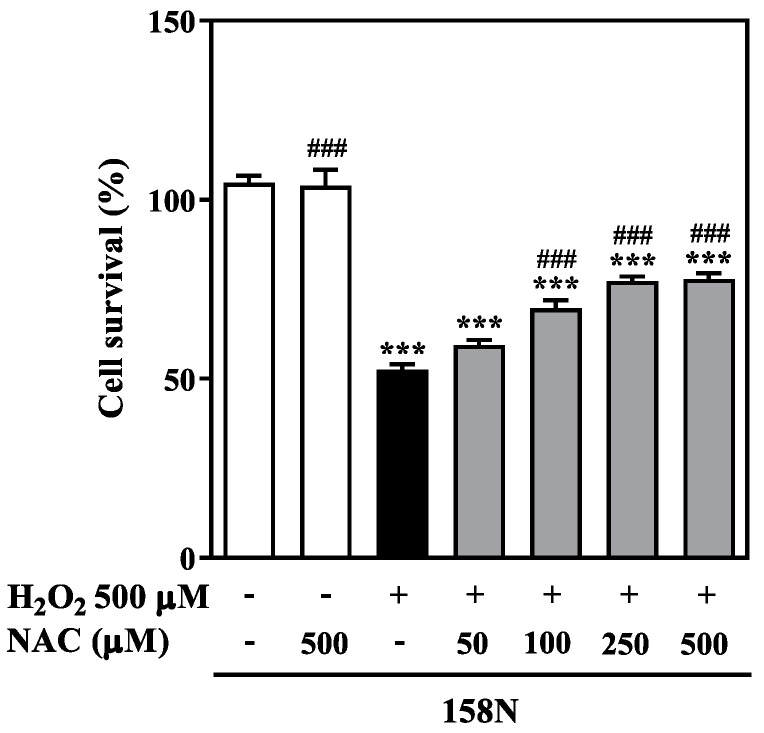
NAC prevents H_2_O_2_-induced oligodendrocyte cell death. 158N cells were treated with H_2_O_2_ (500 µM) for 24 h with and without NAC (50 to 500 µM). Cell survival was calculated as the percentage of control cells, and the results were expressed as mean ± SEM. Cell survival was quantified using a colorimetric method. Data were analyzed by one-way ANOVA with Tukey’s post-hoc test. *** *p* < 0.001 shows significance between control (untreated) and treated groups. ### *p* < 0.001 demonstrates significance between H_2_O_2_ alone and NAC treatment groups in the presence and absence of H_2_O_2_.

We next examined whether the beneficial effect of NAC against H_2_O_2_-induced ROS production could improve survival of 158N oligodendrocytes. Following the exposure of 158N cells to 500 µM H_2_O_2,_ cell survival reduced to 52.3 ± 0.4% relative to controls (Figure 2). However, co-treatment with increasing concentrations of NAC concurrently with H_2_O_2_ exposure increased cell survival in a concentration-dependent manner.

Co-treatment with higher concentrations of NAC (250 µM and 500 µM) and H_2_O_2_ resulted in ~25% increased cell survival (*p* < 0.001). However, lower concentration of NAC (50 µM) did not improve cell survival considerably. Moreover, the significant reduction in ROS observed on treated 158JP cells with NAC (25 µM and 100 µM, Figure 1B) was not associated with further amelioration in cell survival (Figure S1). This is likely because these cells have adapted to the high endogenous ROS levels with a strengthened antioxidant system [21]. These findings demonstrate that NAC is able to protect 158N cells against H_2_O_2_-induced cell death, but did not further improve 158JP cell survival.

### 3.3. GSH Depletion Partially Reduces the Cytoprotective Effect of NAC

**Figure 3:**
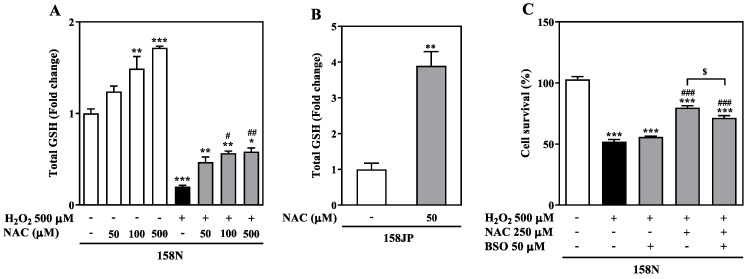
Inhibition of antioxidant glutathione (GSH) synthesis partially blocks the protective effect of NAC. (**A**) Total GSH was analyzed following treatment of 158N cells with NAC (50 to 500 µM) either alone or in combination with H_2_O_2_ (500 µM) for 24 h. (**B**) 158JP cells were treated only with NAC (50 µM) for 24 h. Total GSH was calculated relative to the control (untreated) cells, and results expressed as fold change. (**C**) 158N cells were treated with H_2_O_2_ (500 µM) for 24 h with and without NAC (250 µM) and L-buthionine-(S,R)-sulfoximine (BSO), a GSH synthesis inhibitor (50 µM). Cell survival was calculated as the percentage of control cells and the results were expressed as mean ± SEM. Total GSH levels was analyzed using LC–MS, and cell survival quantified by a colorimetric method. The data were analyzed by one-way ANOVA with Tukey’s post-hoc test (**A**,**C**) and an unpaired Student’s *t*-test (**B**). *** *p* < 0.001, ** *p* < 0.01 and * *p* < 0.05 shows statistical significance between control and treated groups. ### *p* < 0.001, ## *p* < 0.01 and # *p* < 0.05 shows significance between H_2_O_2_ alone and treated groups. $ *p* < 0.05 shows statistical difference between H_2_O_2_ and NAC groups in the presence or absence of BSO.

We explored the mechanism underlying the protective action of NAC against oxidative stress and cell death by measuring the total GSH levels, that includes both reduced and oxidized forms of thiol (–SH+ and –S–S–). Baseline intracellular GSH in 158N cells was observed to be 24.3 ± 1.3 µg/mg total protein, which showed a significant increase in a NAC concentration-dependent manner (Figure 3A). Treatment with 100 µM and 500 µM NAC significantly enhanced the maximal total GSH to 1.5-fold (*p* < 0.01) and 1.7-fold (*p* < 0.001), respectively.

We further evaluated whether NAC can replenish GSH in oligodendrocytes in both constitutive and induced oxidative stress. Incubation with H_2_O_2_ resulted in an 80% reduction in the basal intracellular GSH levels (0.2-fold of control). This depletion of total GSH was partially restored by co-treatment of H_2_O_2_ with 100 µM (*p* < 0.05) or 500 µM NAC (0.6-fold, *p* < 0.01) relative to the H_2_O_2_ group. It is noteworthy that this nominal increase in GSH was associated with an effective increased cell survival by ~ 25% using 500 µM NAC (Figure 2). In order to examine whether NAC can replenish GSH in oligodendrocytes with constitutive high ROS production, we measured the total GSH in 158JP cells exposed to a low concentration of NAC (50 µM). NAC significantly elevated the total GSH to 3.9-fold as compared to controls (*p* < 0.01; Figure 3B). Baseline GSH levels in 158JP cells were 29.1 ± 4.9 µg/mg total protein, which is ~20% higher than in 158N cells, consistent with a strengthened antioxidant system to combat increased ROS.

To further analyze the contribution of GSH to the NAC mechanism of action, we performed inhibitor studies in 158N cells using BSO (50 μM), a well-known GSH synthesis inhibitor [24]. Compared to control cells, treatment with 500 µM H_2_O_2_ decreased cell survival to 50.9 ± 0.6%. This was not further enhanced by addition of BSO to H_2_O_2_ (47.0 ± 1.7%). The supplementation of culture medium with 250 µM NAC significantly increased cell survival by 27.7 ± 0.1% compared to H_2_O_2_ treatment group (*p* < 0.001; Figure 3C). However, this increase in cell survival was partially blocked by the addition of BSO to the above milieu. There was a lower increase in the extent of cell survival in the presence of BSO when compared to NAC plus H_2_O_2_ group (*p* < 0.05) and was only 19.4 ± 0.2% when compared to cells treated with only H_2_O_2_. These results demonstrate that inhibition of GSH synthesis following NAC treatment can partly reduce its cytoprotective effects indicating additional mechanisms contributing to NAC benefits.

### 3.4. HO-1 Activity Mediate the Protective Effect of NAC

**Figure 4:**
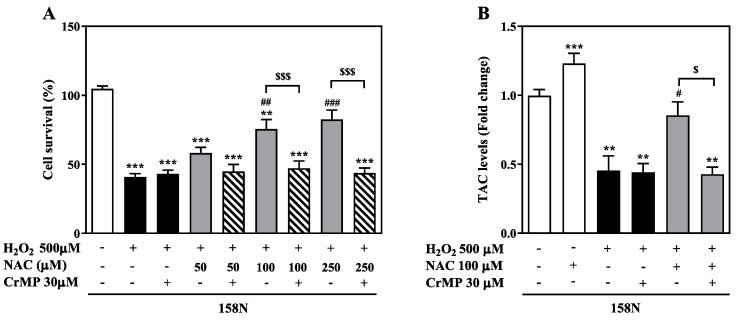
HO-1 activity plays a vital role in the cytoprotective action of NAC. (**A**) 158N cells were incubated with H_2_O_2_ (500 µM) for 24 h with or without NAC (50 to 500 µM) and CrMP (30 µM), a HO activity inhibitor. (**B**) 158N cells were treated for 24 h using different combinations of H_2_O_2_ (500 µM), NAC (100 µM) and CrMP (30 µM). Cell survival (**A**) and total antioxidant capacity (TAC) levels (**B**) were quantified using commercial kits. Cell survival was calculated as the percentage of control cells and the results were expressed as mean ± SEM. TAC levels are represented as relative fold change. The data were analyzed by one-way ANOVA with Tukey’s post-hoc test. *** *p* < 0.001 and ** *p* < 0.01 show significance between control and treated groups. ### *p* < 0.001, ## *p* < 0.01 and # *p* < 0.05 show significance between H_2_O_2_ alone compared to treated groups. $$$ *p* < 0.001 and $ *p* < 0.05 indicate significance between H_2_O_2_ and NAC groups in the presence or absence of CrMP.

To further delineate the mechanisms by which NAC exerts its protective effect, we investigated the role of the inducible antioxidant, HO-1. To this end, we used a selective inhibitor of HO activity, CrMP [25]. As shown previously, increasing concentrations of NAC (50 to 250 µM) with H_2_O_2_ (500 µM) resulted in improved cell survival compared to H_2_O_2_ treated groups. The cell survival rate at 250 µM was similar to untreated cells. However, addition of CrMP (30 µM) along with 100 µM or 250 µM NAC plus H_2_O_2_ resulted in a significant decrease in cell survival in comparison to the same co-treatment without CrMP (*p* < 0.001; Figure 4A).

Treatment with CrMP alone had a slight impact on overall cell survival in comparison to control cells (87.4 ± 2.3%). This finding indicates that the inhibition of HO-1 activity by CrMP led to the loss of the protective effect induced by NAC. Together these results demonstrate that the mechanism underlying the effect of NAC on improving cell survival is primarily mediated by HO-1 activity.

Several studies have demonstrated correlation between the induction of HO-1 activity and increase in total antioxidant capacity (TAC) [26,27]. In order to confirm that CrMP in fact inhibits the downstream antioxidant activity of HO-1, we analyzed the TAC in these experimental conditions (Figure 4B). In this assay, the combined antioxidant activities of vitamins, proteins, lipids, GSH, uric acid, etc. present in cell lysates is compared with Trolox, a water-soluble tocopherol analogue, and was quantified as millimolar Trolox equivalents. The baseline levels of TAC in 158N cells was measured as equivalent to 0.28 ± 0.01 µmol/mg total protein of Trolox. Upon incubation with 100 µM NAC, the TAC increased significantly to 0.34 ± 0.02 µmol/mg total protein of Trolox (1.2-fold of controls, *p* < 0.001; Figure 4B). In contrast, the incubation with H_2_O_2_ decreased TAC levels to 0.13 ± 0.03 µmol/mg total protein of Trolox (0.45-fold of controls), indicating oxidative stress in the cells. The addition of NAC to H_2_O_2_ significantly increased the TAC approximately to baseline levels (0.24 ± 0.03 µmol/mg total protein of Trolox and 0.85-fold of controls; *p* < 0.05). However, addition of CrMP along with NAC and H_2_O_2_ resulted in a significant decrease in TAC (0.12 ± 0.01 µmol/mg total protein of Trolox and 0.43-fold; *p* < 0.05), which is comparable to oxidative stress conditions (H_2_O_2_ treated). Cells treated with CrMP alone had cellular TAC values similar to media controls (0.30 ± 0.006 µmol/mg total protein of Trolox), once again highlighting that a decrease in TAC by CrMP is mediated by HO-1 inhibition. Overall these results are consistent with our observation that the inhibition of HO-1 activity can effectively abolish the antioxidative effects of NAC.

## 4. Discussion

Our study is the first to demonstrate the important role of heme oxygenases in the mechanisms of action of NAC in conditions of oxidative stress in oligodendrocytes. Increasing evidence suggests that the high production of ROS induces toxicity in oligodendrocytes, leading to various diseases of the CNS, including demyelinating disorders [4]. Moreover, oxidative stress has been associated with depletion of GSH resulting in decreased cell survival [11]. Previously we have shown that NAC, an indirect source for GSH, can also increase brain GSH when administered intravenously [12,23]. Constitutive high levels of ROS in mutated glioma cells can be neutralized by a ROS scavenging system dependent on GSH synthesis and metabolism, which can be regenerated by NAC [28]. Similarly, in human astrocytes exposed to organophosphorus insecticide, the replenishment of GSH by NAC prevents cytotoxicity and death, and is correlated with increased levels of ROS and GSH consumption [29]. In another recent study, high exposure to manganese chloride disturbed glutamate-cysteine and cysteine transporters and affected antioxidant content in neurons and astrocytes. It led to the impairment of GSH synthesis and subsequent oxidative stress, both of which were reversed by NAC [30]. Currently, prevention and rescue of oxidative stress by GSH depletion have been considered relevant targets for numerous neurodegenerative diseases.

Here we show that NAC can mitigate oxidative stress resulting from endogenous and exogenous ROS in oligodendrocytes. Additionally, NAC prevented H_2_O_2_-induced cell death in a concentration-dependent manner. Notably, we now show that the cytoprotective property of NAC in conditions of oxidative stress is essentially dependent on the activity of the antioxidant protein, HO-1. HO-1 has been proposed as an inducible and potent protective protein against oxidative stress in neuronal cells exposed to H_2_O_2_ [31], murine models of ischemic stroke [32], and human degenerative and developmental disorders [33]. The neuroprotective effect of HO-1 has been associated to antioxidant and anti-inflammatory activity [18,34]. This is consistent with our observation that HO-1 is a possible target protein of NAC and a mediator of cytoprotective effects in boys with cerebral adrenoleukodystrophy (ALD) [35]. We showed that high dose NAC administered in the setting of hematopoietic cell transplantation in these boys can significantly induce HO-1 expression. Results from the current in vitro study further substantiate this observation. In glioma cells, HO-1 activity is able to support the GSH metabolism by modulating the major glutamate-cysteine transporter [36]. Similarly, in astroglia-like cells ammonia is known to cause oxidative stress, GSH depletion and increased release of pro-inflammatory cytokines. NAC can inhibit ammonia-induced toxicity in these cells, in part by a mechanism dependent on HO-1 activity that further coordinates the downstream inhibition of the NFκB signaling pathway [37].

Neurodegenerative disorders such as PD, AD, multiple sclerosis and ALD are characterized by the selective loss of neurons and progressive CNS dysfunction. Despite the clinical and genetic heterogeneity, these diseases share common pathological mechanisms resulting in neuronal cell death [34,38]. Oligodendrocytes, are a type of glial cells in the CNS crucial for myelin sheath formation [39]. Although the precise mechanism by which demyelination occurs has not been determined, there are reports describing the presence of oxidative stress markers in demyelinating lesions of the patients’ brain. Interestingly, oligodendrocytes have been reported to be the most sensitive cells in the brain to oxidative stress, culminating in cellular damage and death, which contribute to the CNS demyelination process [4]. These reports indicate that mitigating the oxidative stress in oligodendrocytes may have potential therapeutic application in neurodegenerative disorders.

Antioxidants such as NAC, GSH, vitamin E and vitamin C have been evaluated in various models of oxidative stress related to neurodegenerative diseases [40]. NAC is a cell-permeable compound and precursor of L-cysteine which acts as a direct ROS scavenger due its sulfhydryl group. NAC can replenish GSH levels by providing the rate-limiting substrate, L-cysteine [8,41]. GSH is an endogenous non-enzymatic scavenger of ROS and the depletion of GSH is a biomarker of oxidative stress [42]. For instance, low GSH levels were found in the brain tissue of patients with multiple sclerosis [43]. Although oxidative stress has been implicated in ALD, the status of GSH in patients is inconclusive [38,44,45]. Nevertheless, the benefits of high-dose antioxidants including NAC have been investigated in the adult-onset variant of ALD, where normalization of biomarkers of oxidative damage and inflammation was observed [46].

The exact role of HO-1 is still under debate. In spite of that, during stress the upregulation of HO-1 is considered to be an early adaptive event [47]. HO-1 can be induced by a variety of agents including its substrate, heme [48]. Other inducers include heavy metals, heat shock, endotoxin, inflammatory cytokines, and prostaglandins that directly or indirectly generate ROS [49,50]. In addition to oxidative stress related inducers, HO-1 is induced and mediates the antioxidant effects of aspirin [51] and statins [52]. Recently hydrogen sulfide, generated endogenously from L-cysteine, was shown to induce HO-1 in human kidney cells [53]. Interestingly, HO-1 is also found to mediate the antioxidant effects of a variety of antioxidants such as α-lipoic acid [14,54], S-adenosyl methionine [55], curcumin and resveratrol [56], L-methionine [57] and 3-O-caffeoyl-1-methylquinic acid [58].

Here, using an inhibitor, we demonstrate the activity of heme oxygenase to be important for the cytoprotective action of NAC. CrMP can inhibit both HO-1 and HO-2, where HO-2 is a constitutive isoform. NAC has been shown to elevate HO-1 levels through enhancement of Brahma-related gene 1 (Brg1) in cardiac tissues [15]. Similarly, treatment of rat retinal ganglion cells with NAC resulted in increased HO-1 expression during normal redox conditions (~1.5 -fold) [59]. This protective effect was abolished by HO-1 inhibitors and gene-knockouts. However, there are also reports where NAC is used to attenuate HO-1 expression [60,61]. In these studies, the free radical scavenging property of NAC is exploited [62,63]. HO-1 can be induced by free radicals and cellular redox state [64,65]. By removing free radicals, NAC can decrease the expression of HO-1 that was primarily induced by higher ROS levels.

## 5. Conclusions

In this study, we demonstrated the importance of HO-1 as a signaling mediator critical for NAC action in murine oligodendrocytes. NAC reduces ROS, replenishes GSH, as well as protecting 158N cells from cytotoxicity in conditions mimicking oxidative stress. Moreover, in 158JP oligodendrocytes we show that NAC decreases the high intracellular ROS levels by enhancing basal GSH levels. Of note, this antioxidant property was evident in these mutant cells at lower NAC concentrations compared to normal oligodendrocytes, highlighting the potential therapeutic benefit of NAC in genetic conditions causing oxidative stress. In addition to its role as a free radical scavenger, our study shows other important mechanisms of NAC action, which may permit more effective use of this antioxidant in neurodegenerative disorders.

## Supplementary Materials

Supplementary materials can be found at https://www.mdpi.com/2227-9059/8/8/240/s1. Figure S1: Treatment with NAC does not improve 158JP cell survival.
