# Supplementary material for: N-acetylcysteine Provides Cytoprotection in Murine Oligodendrocytes through Heme Oxygenase-1 Activity
Source: Biomedicines. 2020 Jul 23;8(8):240. doi: 10.3390/biomedicines8080240 (PMC7460204; doi:10.3390/biomedicines8080240)
Supplement: Supplementary file 1 [file biomedicines-08-00240-s001.pdf]

N-acetylcysteine Provides Cytoprotection in Murine Oligodendrocytes through Heme Oxygenase-1 Activity

Jie Zhou, Marcia R. Terluk, Lisa Basso, Usha R. Mishra, Paul J. Orchard, James Cloyd, Henning Schröder and Reena V. Kartha

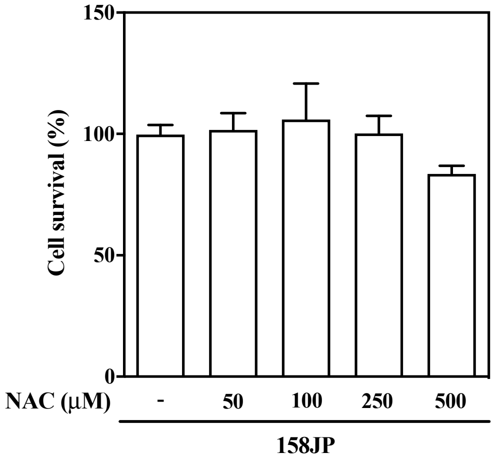

Figure S1 Treatment with NAC does not improve 158JP cell survival. Cells were treated with increasing concentrations of NAC (50 to 500 µM) for 24 h. Cell survival was calculated as the percentage of control cells, and results expressed as mean ± SEM. Data were quantified using a colorimetric method and analyzed by one-way ANOVA with Tukey's post-hoc test.
